# Tight Control of Hypoxia-inducible Factor-α Transient Dynamics Is Essential for Cell Survival in Hypoxia[Fn FN1]

**DOI:** 10.1074/jbc.M113.500405

**Published:** 2014-01-06

**Authors:** James Bagnall, Joseph Leedale, Sarah E. Taylor, David G. Spiller, Michael R. H. White, Kieran J. Sharkey, Rachel N. Bearon, Violaine Sée

**Affiliations:** From the ‡ Centre for Cell Imaging, Institute of Integrative Biology, and; the § Department of Mathematical Sciences, University of Liverpool, Liverpool L69 7ZL, United Kingdom

**Keywords:** Cell Death, Hypoxia, Hypoxia-inducible Factor, Imaging, Mathematical Modeling, Negative Feedback Loop, p53, Prolyl Hydroxylase

## Abstract

Intracellular signaling involving hypoxia-inducible factor (HIF) controls the adaptive responses to hypoxia. There is a growing body of evidence demonstrating that intracellular signals encode temporal information. Thus, the dynamics of protein levels, as well as protein quantity and/or localization, impacts on cell fate. We hypothesized that such temporal encoding has a role in HIF signaling and cell fate decisions triggered by hypoxic conditions. Using live cell imaging in a controlled oxygen environment, we observed transient 3-h pulses of HIF-1α and -2α expression under continuous hypoxia. We postulated that the well described prolyl hydroxylase (PHD) oxygen sensors and HIF negative feedback regulators could be the origin of the pulsatile HIF dynamics. We used iterative mathematical modeling and experimental analysis to scrutinize which parameter of the PHD feedback could control HIF timing and we probed for the functional redundancy between the three main PHD proteins. We identified PHD2 as the main PHD responsible for HIF peak duration. We then demonstrated that this has important consequences, because the transient nature of the HIF pulse prevents cell death by avoiding transcription of p53-dependent pro-apoptotic genes. We have further shown the importance of considering HIF dynamics for coupling mathematical models by using a described HIF-p53 mathematical model. Our results indicate that the tight control of HIF transient dynamics has important functional consequences on the cross-talk with key signaling pathways controlling cell survival, which is likely to impact on HIF targeting strategies for hypoxia-associated diseases such as tumor progression and ischemia.

## Teaser

**Background:** Hypoxia inducible factor-α (HIF-α) is the main transcription factor activated in low oxygen conditions.

**Results:** Single cell imaging reveals pulses in nuclear levels of HIF-α.

**Conclusion:** The transient nature of the HIF-α nuclear accumulation is required to avoid cell death.

**Significance:** The duration of HIF-α response depends on cellular oxygenation, and can encode information and dictate cell fate.

## Introduction

In physiological and/or pathological situations where oxygen homeostasis is lost, the oxygen concentration drops and cells experience hypoxia. The cellular adaptation to hypoxia is mediated at the molecular level by the evolutionary conserved transcription factor hypoxia inducible factor (HIF).^8^ HIF is a heterodimer composed of α and β subunits. The β subunit is constitutively expressed, whereas the main α subunits of HIF, HIF1α and HIF2α, are regulated in an oxygen-dependent manner. Under normoxic conditions, HIFα is hydroxylated, which promotes its binding to the ubiquitin ligase von Hippel-Lindau protein, thereby targeting it for proteasomal destruction (1). However, under hypoxic conditions, HIF-1 and -2α hydroxylation decreases, leading to their rapid accumulation. They then activate the transcription of hundreds of genes encoding proteins involved in cell survival and energy metabolism, but paradoxically also ones involved in apoptosis and autophagy (2). This double-edged sword function of HIF in promoting different cell fates has previously been described and depends on the physiopathological context and differential binding to key partners such as p53 (3). However, the switch from a pro-survival to a pro-apoptotic signal is not well understood. This is an important problem with implications beyond basic biology, because it has direct impact on the management of treatments for solid hypoxic tumors.

We postulated that the temporal regulation of HIF might explain its ability to determine two opposite cell fates. The HIF-VHL and p53-mdm2 signaling systems have previously been shown to share similar network structures in terms of degradation/transactivation loops (4). Furthermore, it has previously been demonstrated, using single cell imaging, that the dynamic behavior of p53 varies, depending on the stimulus, which can influence cell fate decision (5, 6). One important component that can lead to pulsatile or oscillatory behavior is the presence of a negative feedback motif. In the HIF system, prolyl hydroxylase (PHD) -2 and -3 have been proposed as potential delayed negative feedback proteins (7–9). Indeed, PHD1–3 are responsible for HIF hydroxylation, leading to its subsequent degradation (10). Their activity decreases in hypoxia, resulting in HIF accumulation that can in turn activate the transcription of PHD2 and -3. The subsequent PHD increase can compensate for the decrease of activity in prolonged hypoxia and could potentially bring HIF back to low and undetectable levels (7).

We therefore hypothesized that the potential HIF pulse/oscillatory dynamics due to PHD negative feedback could underlie the different cell fate outcomes that have been observed to result from hypoxia. To test this, we have applied a combination of single cell imaging and mathematical modeling. We measured HIF levels in single cells with a high temporal resolution over 20 h. We observed discrete single and repetitive transient pulses of HIF-1α and -2α accumulation when cells were exposed to a hypoxic environment (1% O_2_). We developed a new mathematical model of the HIF-PHD negative feedback loop, which was able to accurately reproduce the single-cell dynamic data, both during a switch from normoxia to hypoxia and during re-oxygenation. We then used the model and experimentation to address the role of the individual PHDs in the generation of HIF dynamics. This demonstrated an essential role for PHD2 in the control of the transient dynamics of HIF and in the prevention of cell death triggered by long lasting HIF-1α levels. Changes in HIF dynamics and levels will likely affect directly HIF transcriptional activity, as well as the activity of its binding partners, for example, mdm2 (11). Indeed the effects of PhD2 silencing on cell death were correlated with a strong transcription of p53-dependent pro-apoptotic genes in hypoxia. Moreover, coupling of HIF and p53 mathematical models (12) predicted significant effects of the variations of HIF dynamics on the oscillations and levels of the p53 protein, indicating that HIF dynamics not only affects HIF signaling but also its cross-talk with other essentials signaling systems involved in the control of cell fate.

## EXPERIMENTAL PROCEDURES

### Reagents and Antibodies

Tissue culture medium was from Invitrogen; fetal calf serum (FCS) from Harlan Seralab (UK); and pharmacological inhibitor dimethyloxaloylglycine from Alexis Biochemicals (Enzolife Sciences, New York). Cycloheximide was from Calbiochem (Merck, Darmstadt, Germany). The antibody against HIF-1 was from BD Biosciences (catalog number 61-0959) and the antibody against HIF-2 was from Santa Cruz Biotechnology (catalog number Sc 13 596). EGFP antibody was from Abcam (catalog number ab290).

### Cell Culture and Hypoxia

HeLa cells were grown in Dulbecco's modified Eagle's medium (DMEM) supplemented with 10% FCS (v/v) and 1% nonessential amino acids (v/v), at 37 °C, 5% CO_2_. Cells (between passages 8 and 20) were plated at 1 × 10^5^ cells/ml. shPHD2 HeLa cells (generous gift from D. Hoogewijs, D. Stiehl, and R. Wenger, University of Zürich, Switzerland) were grown in the same medium as WT HeLa supplemented with 10 μg/ml of puromycin for maintaining the hairpin expression. The C51 colon adenocarcinoma cells pH3SVL (generous gift from S. Lehmann and R. Wenger, University of Zürich, Switzerland) have a stably integrated plasmid containing a minimal SV40 promoter regulated by 3 HREs from the human transferrin promoter. There were grown in DMEM high glucose, 10% FCS (v/v), and 400 μg/ml of G418. The ODD-EGFP HeLa cell line was generated by transduction of a HIV-ODD-EGFP-ires dTomato lentivirus. For imaging experiments, cells were plated in 35-mm glass bottom dishes (Iwaki, Bibby Sterilin, UK). Hypoxic incubation was performed either directly onto the microscope stage equipped with a PeCon incubator with an O_2_ controller unit or in a hypoxic work station (Don Whitley Scientific, England) for bulk cell experiments (1% O_2_, 5% CO_2_, 94% N_2_).

### Immunoblotting

Total protein was extracted with a lysis buffer (50 mm Tris-HCl, pH 7.5, 1 mm EDTA, 1 mm EGTA, 1% (v/v) Triton X-100, 50 mm NaF 50, 5 mm sodium pyrophosphate, 10 mm sodium β-glycerophosphate, 0.1 mm PMSF, 1/100 protease inhibitor mixture, and 1/100 phosphatase inhibitor mixture). After 1 h at 4 °C on a rotating wheel shaker, the lysates were centrifuged at 10,000 × *g* for 15 min at 4 °C and total protein concentration was measured with BCA assay in the supernatant. 40 μg of proteins were resolved by SDS-PAGE (10% gels) and transferred onto nitrocellulose membrane. The membranes were blocked with 5% nonfat dry milk in TBS-T (10 mm Tris-HCl, pH 8, 100 mm NaCl, 1% (v/v) Tween 20) and incubated with appropriate primary antibody (overnight, 4 °C), followed by incubation with horseradish peroxidase-conjugated secondary antibody (1 h at RT). SuperSignal West Dura Extended Duration Chemiluminescent Substrate was used for the ECL reaction and the signal was detected and quantified using the G:box gel doc system (Syngene, UK).

### Quantitative RT-PCR (qPCR) and Primers

Cellular RNA was purified using Qiagen RNeasy mini kit according to the manufacturer's instructions. cDNA was synthesized with a QuantiTect Reverse Transcription Kit and qPCR was performed using ABI Power SYBR Green PCR master mix according to the manufacturer's instructions. We used an ABI 7500 Fast Real-time PCR System. Cyclophilin A was used as a calibrator for the relative amplification of genes of interest calculations. Primer sequences used were: cyclophilin A forward, GCTTTGGGTCCAGGAATGG, reverse, GTTGTCCACAGTCAGCAATGGT; PHD2 forward, GGAAGATGGAGAACCTGCTG, reverse, GCTTGTGCTTCTTCCAGTCC; PHD3 forward, AGATCGTAGGAACCCACACG, reverse, TTCTGCCCTTTCTTCAGCAT; PHD1 forward, ACTGGGACGTTAAGGTGCAT, reverse, AAATGAGCAACCGGTCAAAG; VEGF forward, TCTTCAAGCCATCCTGTGTG, reverse, ATCTGCATGGTGATGTTGGA; Puma forward, CTTGGAGGGTCCTGTACAAT, reverse, CACCTAATTGGGCTCATCT; and Noxa forward, CGAAGATTACCGCTGGCCTA, reverse, ATGTGCTGAGTTGGCACTGA.

### Gene Transfer

#### 

##### Plasmids

Fluorescent HIF-1 and -2α fusion constructs were cloned in the Gateway system (Invitrogen). HIF sequences were amplified by PCR using a plasmid template and cloned into a Gateway Entry vector by recombination. The final EGFP fusion was obtained by recombination of the HIF-entry vector with a EGFP destination vector. PHD1-EGFP and PHD3-EGFP were obtained from the Addgene non-profit making plasmid repository (catalog number plasmids 21400 and 21402), both plasmids were described in Ref. 13. PHD2-EGFP was a generous gift of Dr. R. Depping (University of Lübeck, Germany). pPHD2-PHD2-EGFP was constructed by replacing the CMV promoter of the PHD2-EGFP plasmid by 1 kb of the PHD2 promoter (amplified from a Bacterial Artificial Chromosome template from Invitrogen).

##### Transfection

Cells were transfected 24 to 48 h before imaging using FuGENE 6 (Roche Applied Sciences, UK) according to the manufacturer's instructions with a FuGENE/DNA ratio of 2/1.

##### Lentivirus

ODD-EGFP lentiviral transfer vectors were produced by insertion of the fusion of human HIF-1α ODD (amino acids 529–652)-EGFP, amplified from previously made gateway plasmid pG-ODD-EGFP into the lentivector pHIV-ires-Tomato (Addgene plasmid 21374). The shPHD3 lentivirus was obtained from D. Hoogewijs and R. Wenger (University of Zurich). pMD2.G (Addgene plasmids 12259 and Addgene plasmid 12260) was used for packaging.

##### Viral Transduction

Lentiviral particles were produced by transfection of the 293TN cell line using calcium chloride. The medium was replaced 16 h post-transfection and collected 24 h later, cleared by low speed centrifugation, and filtered through a 0.45-μm pore filter. After ultracentrifugation on 20% sucrose, the virus pellet was re-suspended in 200 μl of PBS. A serial dilution of concentrated virus was used to transduce HeLa cells in the presence of Polybrene (8 μg/ml).

### Time Lapse Confocal Microscopy

Cells were incubated on the microscope stage at 37 °C, 5% CO_2_, 1 or 20% O_2_ and observed by confocal microscopy using a Zeiss LSM510 with a Plan-apochromat ×63 1.3 NA oil immersion objective. Excitation of EGFP was performed using an argon ion laser at 488 nm. Emitted light was detected through a 505–550 nm bandpass filter from a 545-nm dichroic mirror. Excitation of the empty dsRed used as a control was performed using a green helium-neon laser (543 nm) and detected through both a 545-nm dichroic mirror and a 560-nm long pass filter. Data capture was carried out with LSM510 version 3 software (Zeiss, Germany) using the Auto-time series macro (14). For time lapse experiments mean fluorescence intensity was extracted and the fluorescence intensity was determined for each cell using CellTracker version 0.6 software (15). These experiments were performed three times and ∼100 cells were analyzed for each HIF-1α and -2α construct. For promPHD2-PHD2-EGFP, the experiment was performed three times and ∼50 cells were analyzed.

### Imaging Analysis

For analysis, cells were always co-transfected with an empty dsRed plasmid to monitor transfection in normoxia as well as normalize fluorescence levels over time. Only cells visibly transfected with a dsRed-expressed control plasmid were analyzed. A region of the nucleus was followed by CellTracker and the data exported as mean intensity of fluorescence. Cells that were clearly transfected with the empty red plasmid and which showed a change in green fluorescence, but not in red fluorescence, were scored as responsive. The fluorescence intensity data were then averaged by calculating the mean of 10 consecutive time points. A threshold technique was used for characterization of the response time and response duration. This threshold was calculated for each cell, and was defined as the 50% value between maximum and minimum fluorescence intensity. Cells that died or migrated out of the recorded field within the first 4 h of the experiment were not analyzed. Cells that died less than 3 h after a HIF-EGFP increase were also removed from the analysis. Cell death was monitored on bright field images. For classification between transient, prolonged, and multiple peak response, an automatic peak detection was implemented. The threshold, calculated as described previously, was additionally scaled to the maximal amplitude and standard deviation. Cells with a response shorter than 280 min were considered as transient. Cells with multiple threshold crossing were classified as multiple responders.

### Annexin V/PI Labeling

Apoptosis was assessed by addition in culture medium of propidium iodide (PI) to 0.5 μg/ml and annexin V-FITC (Sigma) to 1.0 μg/ml. Images were taken every 15 min through a ×20 objective. Excitation was at 488 nm for PI and fluorescein. PI fluorescence was collected through a 560-nm long-pass filter and FITC from a 505–530 nm bandpass filter.

### Statistical Analysis

Statistical significance was determined by one-way analysis of variance followed by a Bonferroni multiple comparison test. Difference was considered as significant at *p* < 0.01. All the experiments were performed at least 3 times.

### Luminescence Microscopy

Luciferin was added (0.5 mm, Biosynth AG, Switzerland) to 3 ml of medium containing cells in 35-mm glass coverslip culture dishes (Iwaki), and incubated on the microscope stage at 37 °C, 5% CO_2_, and 20 or 1% O_2_. Imaging was carried out using a Zeiss Axiovert 100 microscope with a Fluor 10 × 0.5 NA objective. The photons emitted by individual cells were collected using a Hamamatsu ORCAII BT 512 CCD camera (C4742-98 Hamamatsu Photonics Ltd, UK) controlled with Metamorph software. A series of images were acquired using a 30-min integration time over 80 h. AQM advanced 6 software (Kinetic Imaging, UK) was used for image analysis with background correction. All these experiments were performed at least three times and in each experiment at least 30 cells were recorded and analyzed.

### Flow Cytometry

Cells were seeded in 6-cm dishes at a total density of 100,000 cells and co-transfected with HIF1α-EGFP and dsRED-XP, 1 day before hypoxic incubation as indicated. Upon hypoxic incubation, cells were trypsinized and pelleted and then resuspended in 100 μl of PBS. 100 μl of 4% paraformaldehyde was added (final concentration of 2%) and incubated for 15 min at room temperature. Hypoxic samples were fixed in the hypoxic chamber. Analysis was carried out using a Guava EasyCyte Plus Flow Cytometer (Millipore). The percentage of EGFP and dsRed positive cells in each sample was established using GuavaSoft software (Millipore).

### Mathematical Modeling

Fig. 3*A* describes the minimal model consisting of two coupled ordinary differential equations: *HIF*-1α (*x*) is produced through basal synthesis at rate *S*, induces the transcription of PHD (*y*) at rate *k*, and is degraded via PHD-dependent hydroxylation at an oxygen-dependent maximal rate *h* with saturation threshold, γ. All models were solved in Matlab R2009a using standard ordinary differential equation solvers. Parameters in the two-component model were optimized for each cell time series data by minimizing the sum of squared residuals of the ordinary differential equation solution and the experimental data using the built-in Matlab function *fminsearch*. The ratio of hydroxylation rate in hypoxia to normoxia was taken to be 0.14 based on measured values from the literature for the PHD2 isoform (16). Initially free parameter optimization was performed on bell-shaped single-cell data, and median values were obtained. Parameter optimization was then constrained so that *k* and *d* could only vary from the median values by 50%. Parameters S and γ were unconstrained as experimental protocols such as transfection efficiency or laser intensity may result in variability between cells. Fits were classified as good or bad using an error envelope defined by EXP(*t*) ± 0.35(max(EXP(*t*)) − min(EXP(*t*))), where EXP(*t*) represents the time series vector of experimental data. Solutions were classified as bad fits if more than 1% of the experimental data points lay outside the error envelope and good fits otherwise.

The two-component model was extended to distinguish PHD1 (*y*_1_), PHD2 (*y*_2_), and PHD3 (*y*_3_).

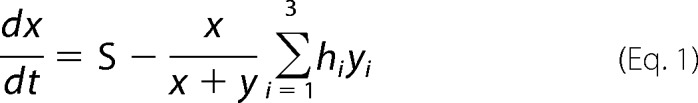


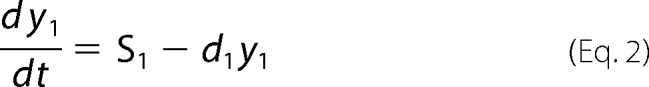


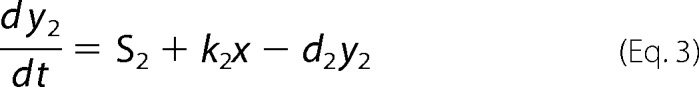


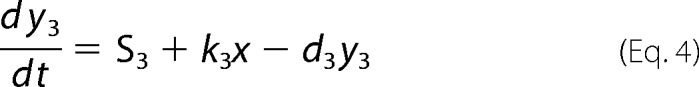
 PHD2 and PHD3 are HIF inducible with induction rates *k*_2_ and *k*_3_. The PHD basal degradation parameters, *d_i_* (*i* = 1,2,3) were taken to be the mean values of measured half-lives (Fig. 5*B*). The hydroxylation rate parameters, *h_i_* (*i* = 1,2,3), were based on measured values from the literature (16). In the extended model, it was necessary to introduce basal synthesis of the PHD proteins, as PHD1 is not produced via HIF induction. To estimate the PHD basal synthesis rates, *S_i_*, the steady state ratio of proteins were taken to be 0.2:0.8:0.1, based on data from Ref. 17. Free parameters were optimized by fitting the 4-component model solution to a median cell generated from the 2-component model optimization of de-oxygenation data.

The HIF-PHD model was coupled to a previous model describing the p53-Mdm2 feedback loop (12). In the original p53-Mdm2 model, HIF binds to p53 reducing the rate at which p53 is degraded when in a complex with Mdm2. In the original model this is represented by a switch in the degradation rate following hypoxic stress. Here the model is extended to allow the degradation rate to be explicitly a function of HIF, δ(*x*) = *Ae*^−*Bx*^, with constants *A* and *B* chosen so that the degradation rate matches the original model when the HIF levels have attained equilibrium steady state values in normoxia (20% oxygen, low HIF) or hypoxia (1% oxygen, high HIF). Model simulations were initially run with HIF switching between equilibrium levels (“steady HIF dependence”) to recapitulate the results of Hunziker *et al.* (12). The model was then coupled to the four-component HIF-PHD model (“dynamic HIF dependence”).

## RESULTS

### 

#### 

##### Single Cell Dynamics of HIF-1α and -2α in Normoxia and Hypoxia

**FIGURE 1.**
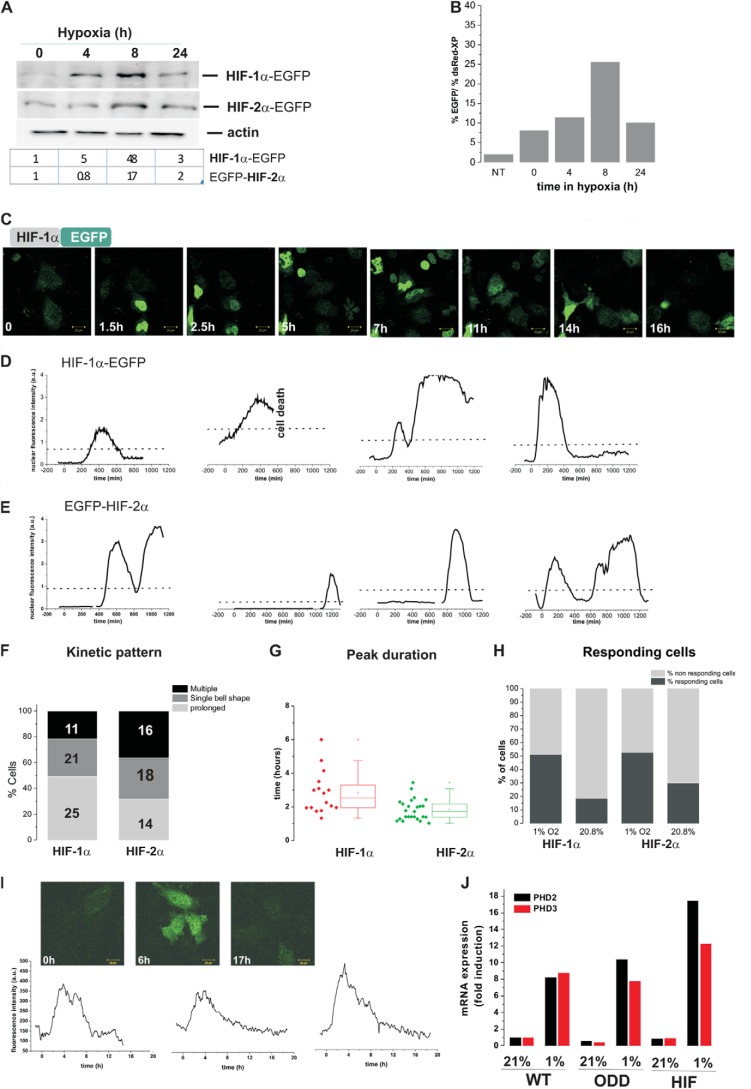
**Single cell dynamics of HIF-α nuclear levels and HIF-dependent transcription.** *A*, HeLa cells were transfected with HIF-1α-EGFP or EGFP-HIF-2α. 24 h after transfection cells were exposed to hypoxia (1% O_2_) for the indicated time points. HIF-EGFP fusion protein levels were assessed by Western blot using an anti-GFP antibody and the bands were quantified by densitometry analysis. *B*, HeLa cells were transfected with HIF-1α-EGFP together with dsRED-XP expression plasmid for normalization purposes. % of red and green fluorescent cells were measured by flow cytomtery and plotted as a function of time in hypoxia. Nontransfected controls (*NT*) were used for gating. *C,* selected representative images of HeLa cells transiently co-transfected with HIF-1α-EGFP and an empty dsRED Express control plasmid (not shown on the picture) to monitor the localization and number of transfected cells. Transfection efficiency was ranged from 30 to 40%. Cells were imaged using time lapse confocal microscopy every 5 min in 20.8% O_2_ for 1 h and then switched to 1% O_2_ for 20 h. See also supplemental Movies S1 and S2. *D* and *E*, nuclear fluorescence levels for HIF-1α (*D*) and HIF-2α (*E*) were plotted as a function of time for 4 representative cells. The *straight line* represents the threshold used for automatic peak detection (see “Experimental Procedures”). All traces for HIF-1α are shown in supplemental Fig. S1*A*. Some traces are shorter than the entire time course due to either cell death or migration out of the imaging field. *F*, classification of the observed HIF-α response kinetics. Transient bell shapes curves and multiple peaks were scored using a threshold (see “Experimental Procedures”). The number of cells scored in each category are indicated on the plot. *G*, duration of the HIF accumulation in transient response. Duration was determined as the time between the point at passing half-maximum fluorescence and returning below this value, the 25th to 75th quintile is indicated on the plot. *H,* percentage of transfected cells showing an increase of green fluorescence levels in hypoxia and normoxia. *I*, a stable HeLa cell line expressing the HIF-1α ODD-dependent ODD from residues (amino acids 529–652) fused to EGFP was generated by lentiviral transduction of a HIV-ODD-EGFP-ires-dTomato vector into HeLa cells. The ODD cell line was imaged in normoxia before a switch to 1% O_2_ for 20 h. Fluorescence intensities were quantified and plotted as a function of time. Four representative plots are shown, 50 cells were tracked in total, 82% displayed pulsed dynamics. *J*, HeLa cells were transiently transfected with HIF-1α-EGF, either stably expressing ODD-EGFP or left non-transfected. They were cultured in normoxia or hypoxia (1% O_2_) for 8 h prior to cell lysis and mRNA extraction. mRNA levels for PHD2 and -3 were measured by qPCR and normalized to cyclophilin A mRNA.

**FIGURE 2.**
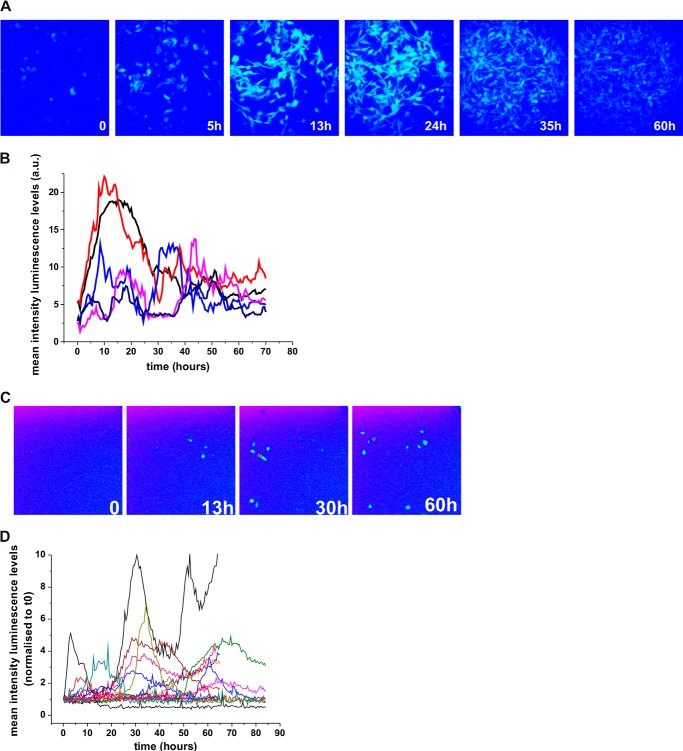
*A,* luminescence images of C51 cells stably transfected with HRE-luciferase (PH3SVL cells) in 1% O_2_. Luminescence levels were imaged using wide field microcopy in the presence of luciferin in cell culture in 1% O_2_ for 80 h. *B,* mean luminescence levels of single cells were plotted as a function of time. Each *color line* is a representative cell. *C,* HeLa cells were transiently transfected with a HRE-luciferase reporter vector. *D,* luminescence levels in hypoxia were acquired and quantified as in *B*.

To capture HIF-1α and HIF-2α dynamics, we used time lapse confocal imaging of HIF-EGFP (enhanced green fluorescent protein) fusion proteins in an O_2_ controlled environment. HIF-1α-EGFP and EGFP-HIF-2α induction in hypoxia was validated by EGFP detection (Fig. 1*A*). In cells switched from a normoxic to a hypoxic environment, we observed by Western blot (Fig. 1*A*) and flow cytometry (Fig. 1*B*), a HIF-1α-EGFP accumulation at 4–8 h that had decayed by 24 h, which was in agreement with previously published results on endogenous HIF (7, 18, 19). HIF-2α was less inducible by hypoxia, and was already detectable in normoxia. For live-cell imaging, observations were initially taken at 20.8% O_2_ tension as a control for 2 h, then, after a switch from 20.8 to 1% O_2_ cells were further imaged for 20 h. Cells subjected to the hypoxic switch showed transient HIF-1α and -2α nuclear accumulation with varied kinetics (Fig. 1, *C–E*, supplemental Fig. S1*A* and Movies S1 and S2). A single transient bell-shaped profile was observed in 30% (21 cells) of the HIF-1α and 32% (18 cells) of HIF-2α responding cells (Fig. 1*F*), which was of similar duration, ∼2–4 h; Fig. 1*G*). We observed some spontaneous pulses of HIF-1α nuclear accumulation in normoxic cells (19% of the transfected cells, Fig. 1*H*). These levels were higher for HIF-2α (30% of the transfected cells), consistent with the stabilization observed by Western blots in normoxic conditions. We observed that 22% of HIF-1α (11 cells) and 36% of HIF-2α cells (16 cells) had more than 1 peak of HIF nuclear accumulation. In most cases the subsequent peaks had increased amplitude (Fig. 1, *D–F*). The transient pattern of HIF dynamics was also observed in other cell lines (*e.g.* HEK293T cells, not shown) and also observed in a HeLa cell line stably expressing the oxygen degradation domain (ODD) of HIF-1α fused to EGFP (Fig. 1*H*). Cells visibly expressing ODD-EGFP displayed similar transient dynamics, yet contrary to HIF-1α-EGFP the degradation was slower. This is likely due to the ODD not possessing transcriptional activity and so being unable to further increase the PHD feedback above endogenous regulation, in contrast to full-length HIF exogenous expression (Fig. 1*I*). We also characterized the transient dynamics of HIF activity. The HIF-dependent transcriptional activity in live cells was assessed by imaging the light produced by a hypoxia response element-luciferase reporter gene (*HRE-luc*). We examined C51 cells stably expressing a HRE-luciferase (PH3-SVL C51) (Fig. 2, *A* and *B*) and HeLa cells transiently transfected with HRE-luc (Fig. 2, *C* and *D*). In both conditions, we found a transient luciferase signal, indicating transient transcriptional activity. Some cells displayed 2 or more peaks of luciferase expression, which is consistent with the nuclear accumulation of HIF fluorescent fusion proteins. Interestingly, the stable C51 cells had some basal luminescence signal in normoxia, indicating some degree of spontaneous low amplitude luciferase peaks of transcriptional activity (not shown) in agreement with the observation of spontaneous accumulation of HIF-1α and -2α.

##### Mathematical Modeling of HIF Nuclear Dynamics during Hypoxia and Re-oxygenation

**FIGURE 3.**
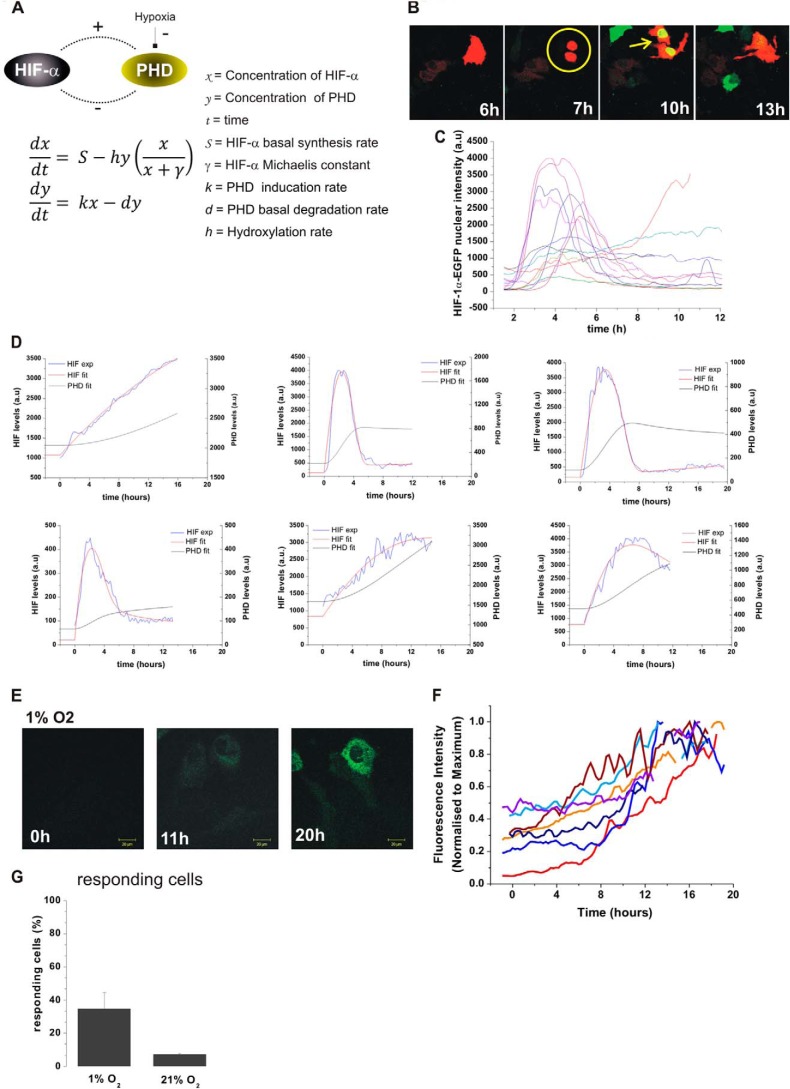
**Mathematical modeling of the generic HIF-PHD feedback loop.** *A,* description of the model (see “Experimental Procedures”). HIF-α (*x*) is produced at rate *S* and removed due to PHD (*y*) hydroxylation. The maximal hydroxylation rate, *h*, is oxygen dependent and saturation of hydroxylation is determined by the parameter γ. PHD (*y*) is produced through induction by HIF-α at rate *k* and undergoes basal degradation at rate *d. B,* HeLa cells were transiently co-transfected with HIF-1α-EGFP and an empty dsRED-Express control plasmid. Cells were imaged every 5 min after a switch from 20 to 1% O_2_ using time lapse confocal microscopy. The pictures show a typical example of HIF-1α nuclear accumulation occurring after cell division. This was observed in 50% of the cells showing a HIF-1α increase. *C,* HIF-1α levels in hypoxia plotted as a function of time, synchronized based on cell cycle. *D,* single cell data of HIF dynamics (*blue line*) were fitted computationally using the model (*red line*). See also supplemental Fig. S1 for more cell fitting. The model PHD profile is in *green*. The model cells are initially at equilibrium in normoxia (*h* = 1) and are de-oxygenated into hypoxia (h = 0.14) at *t* = 0. *E*, selected representative images of HeLa cells transiently transfected with the PHD2prom-PHD2-EGFP expression plasmid. Cells were imaged using time lapse confocal microscopy every 5 min in 20.8% O_2_ for 1 h and then switched to 1% O_2_ for 20 h. *F,* the plots represent the whole cell fluorescence intensity produced from PHD2prom-PHD2-EGFP as a function of time. *G*, the percentage of responsive cells is calculated from the number of transfected cells showing an increase of the green fluorescence level over time from PHD2prom-PHD2-EGFP in hypoxia and in normoxia.

The known negative feedback from PHD proteins on HIF is a likely candidate for generating the pulses of HIF-1α and -2α (9, 10, 20). To analyze the dynamic behavior of HIF, we developed a simple mathematical model based on the global HIF-PHD negative feedback loop (Fig. 3*A*). We observed heterogeneity in the response time and that HIFα responses occurred after cell division in 50% of dividing cells (see Fig. 3*B* for a typical example). This might have been caused by transient transfection at the time of nuclear breakdown (21). Cells were, therefore, artificially synchronized in the cell cycle (Fig. 3*C*), using the mitosis time as *t* = 0 for mathematical modeling.

We initially fitted the bell-shaped single-cell data (from HIF-1α-EGFP)), which encapsulate more complex dynamics and better mirrors the transiency of accumulation observed by Western blot, by using models previously described for the p53 system (22). A model, which included a saturation coefficient for hydroxylation provided the best fit (Fig. 3*D*, and “Experimental Procedures”). 79% (31/39) of the de-oxygenation single cell data were then successfully fit to the model, subject to the constraint that the parameters *k* (induced PHD production rate) and *d* (PHD degradation rate) were similar across all cells (see supplemental Fig. S1A and “Experimental Procedures”). The model predicted a slow gradual increase of PHD. This was in qualitative agreement with the PHD2 dynamics measured in single cells using PHD2-EGFP controlled by the PHD2 proximal promoter (Fig. 3, *E* and *F*); PHD2 up-regulation was observed in 40% (20 cells) of the transfected cells switched to 1% O_2_ (Fig. 3*G*).

**FIGURE 4.**
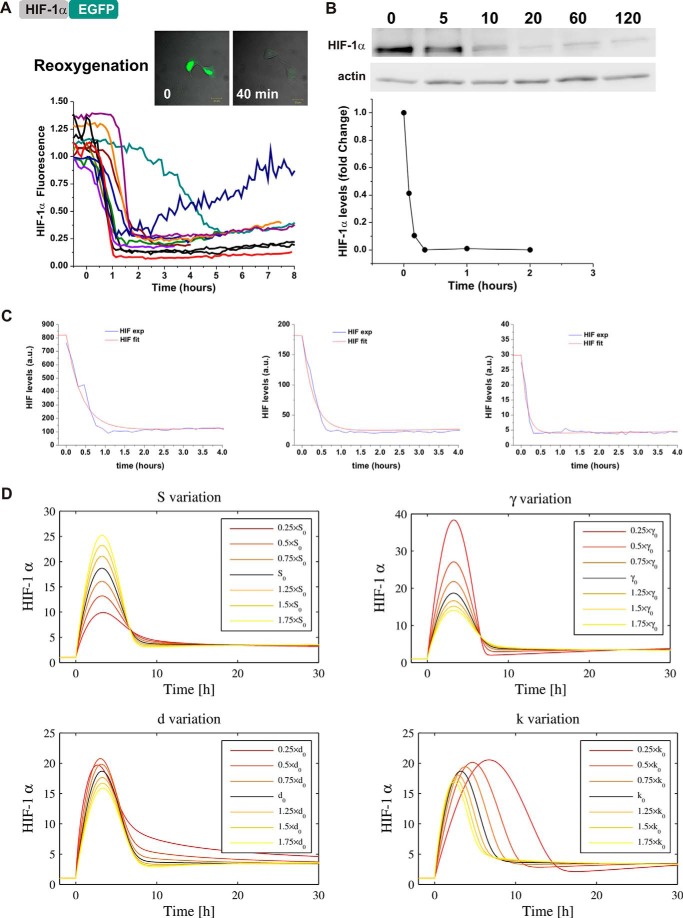
**Single cell dynamics during re-oxygenation and validation of the mathematical model.** *A*, cells were transfected as described in the legend to Fig. 1*B*. 24 h after transfection, cells were exposed to 1% O_2_ for 6 h in the microscope stage and then re-oxygenated to 20.8% O_2_. Fluorescent levels were measured 1 h prior and during the re-oxygenation period and plotted as a function of time. *B,* HeLa cells were cultured in hypoxia for 6 h and then subjected to re-oxygenation. HIF-1α levels were measured by Western blot at the indicated time points. Densitometry analysis of the bands were plotted as a function of time. *C*, using the model described in the legend to Fig. 2, we fitted the single cell traces obtained experimentally in *A*. The cells are initially at equilibrium in hypoxia (h = 0.14) and are re-oxygenated back into normoxia (h = 1) at *t* = 0. *D,* parameter sensitivity analysis was conducted by varying one parameter at a time, as a perturbation from the artificial cell obtained from the median parameters (*S* = 2.38 × 10^1^ AU min^−1^, γ = 2.98 × 10^2^ AU, *k* = 4.71 × 10^−4^ min^−2^, *d* = 4.71 × 10^−4^ min^−1^]). Pre-stimulation (normoxic) equilibria have been normalized to 1 to emphasize the qualitative effects of parameter variation. The *black curve* represents the median cell model output and parameters were individually deviated either way in steps of 25% of the median value varying up to ±75%.

We further assessed the functionality of the experimental system and the model by fitting re-oxygenation experiments. Therefore, HIF-1α levels were imaged in single cells during re-oxygenation after exposing cells to hypoxia for 6 h. Upon re-oxygenation, HIF-1α-EGFP-transfected cells displayed a rapid loss of fluorescence, presumably due to its degradation (Fig. 4*A*). The kinetics were slower, but consistent with the endogenous HIF-1α degradation observed by Western blot (Fig. 4*B*), supporting the validity of the experimental imaging results. Interestingly, in some cells, the loss of fluorescence was not definitive and these cells had a clear slow return of fluorescence 200 min after re-oxygenation. We fitted all cells obtained from the re-oxygenation experiment with the parameters *k* and *d* constrained and good fits were obtained for 74% (31/42) of cells (example of 3 cells shown in Fig. 4*C*, all cells in supplemental Fig. S1*B*).

##### Role of PHDs in HIF Timing and Transiency

**FIGURE 5.**
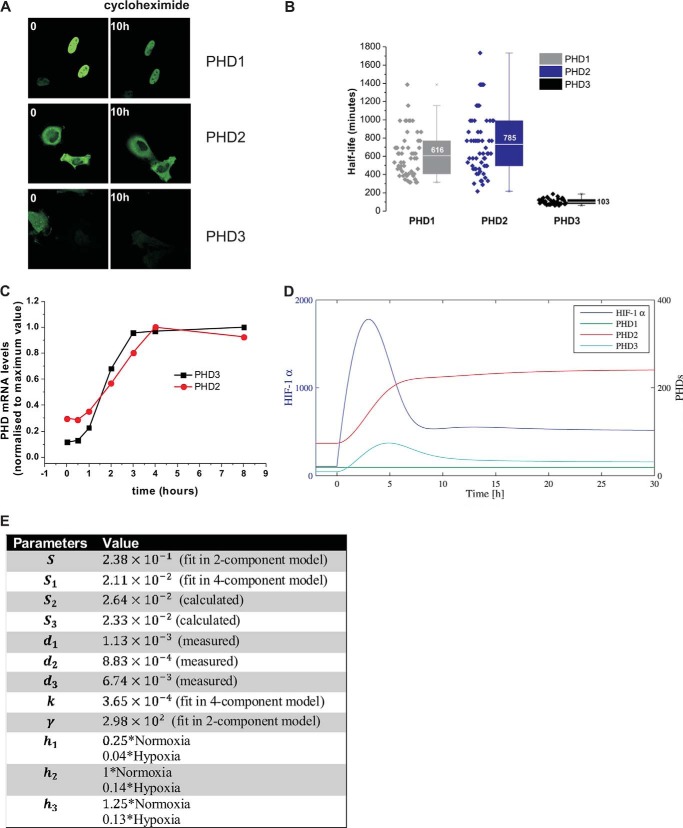
*A*, measurement of the half-life of PHD1, -2, and -3. HeLa cells were transfected with PHD1, -2, -3-EGFP. 24 h after transfection, cells were treated with cycloheximide (10 μg/μl) and the PHDs levels were monitored for up to 24 h by measuring fluorescence intensity. *B,* box and whisker plot of the half-life measured in single cells for PHD1, -2, and -3. *C,* qPCR analysis of PHD2 and PHD3 mRNA induction during a hypoxic time course (1% O_2_). Each time point sample was generated in triplicates. The plot is representative of one experiment. The experiment was repeated 4 times. *D*, four component model run. The model cell is initially at equilibrium in normoxia and is then de-oxygenated into hypoxia at *t* = 0. *E,* the parameter sets for the 4-component model are detailed in the table (time units in min).

**FIGURE 6.**
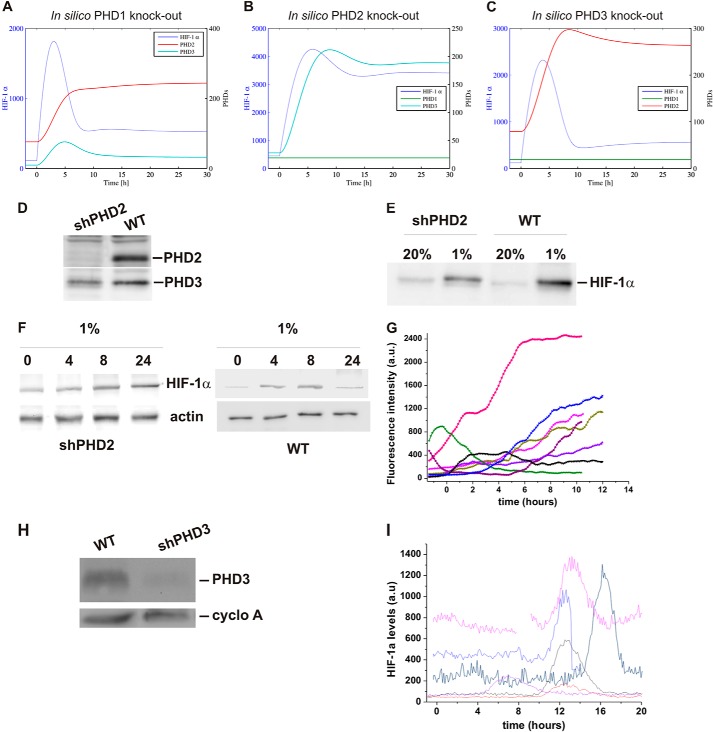
**PHD2 knock-down affects HIF temporal profile.** *A–C*, predictions are based on a 4-component model (Fig. 5*A*) with PHD1, -2, or -3 removed representing *in silico* knock-out of PHD1, -2, and -3. The models are initially at equilibrium in normoxia and then de-oxygenated into hypoxia at *t* = 0. *D,* Western blot analysis of PHD2 and PHD3 levels in WT and sh-PHD2 HeLa cells. *E,* Western blot analysis of HIF-1α levels in WT and SH-PHD2 HeLa cells cultured in normoxia submitted to 5 h hypoxia (1% O_2_). *F*, Western blot analysis of HIF-1α levels in sh-PHD2 HeLa cells cultured in 1% O_2_ for the indicated time points. *G,* single cell analysis of HIF-1α-EGFP levels in sh-PHD2 cells exposed to 1% O_2_. *H,* Western blot analysis of PHD3 levels in WT and sh-PHD3 HeLa cells. *I,* single cell analysis of HIF-1α-EGFP levels in sh-PHD3 cells exposed to 1% O_2_.

A generic “median cell” based on median parameter values was constructed and used to test the effects of parameter variation (Fig. 4*D*). Varying S (basal synthesis rate of HIF-1α) had a clear effect on the amplitude, but not on the kinetics, of the response. This could explain the difference in amplitude observed in the single cells (visible in Fig. 3*C*), which are likely to have different copy numbers of HIF plasmid due to transfection variability. In contrast, varying *k* (HIF-dependent PHD induction) affected the duration of HIF accumulation. Therefore, we decided to test computationally the potential redundancy of the PHD feedback in HIF temporal regulation. The model describes a generic PHD, which is in reality a combination of PHD2 and PHD3 (HIF-inducible) and PHD1 (non HIF-inducible). We introduced a new level of complexity, by separating the 3 different PHDs (“Experimental Procedures”), which may have different induction rates and protein stabilities (*k* and *d* parameters, respectively). Imaging experiments using cycloheximide to block protein synthesis showed that PHD2 and -1 were stable with a half-life of more than 10 h, whereas PHD3 was less stable with a half-life of 1.7 h (Fig. 5, *A* and *B*). Based on our measurements of mRNA production in hypoxia, we estimated the induction rate (*k*) of PHD2 and PHD3 to be similar (Fig. 5*C*). The model was run to equilibrium and a switch to hypoxia was applied (Fig. 5, *D* and *E*). In WT cells, the equilibrium value of PHD2 was significantly higher than the other isoforms, because the model was based on the steady state ratio of proteins to be 0.2/0.8/0.1 for isoforms PHD1:PHD2:PHD3 based on (17). Furthermore, PHD2 takes longer than PHD3 to stabilize to an equilibrium level, which can be explained because PHD2 degrades more slowly than PHD3. *In silico* knockdown of PHD1 and PHD3 had little effect on HIF pulse duration. In contrast, removal of PHD2 led to a sustained HIF stabilization (Fig. 6, *A–C*), indicating that this was the most important factor in the control of HIF dynamics. We then tested experimentally the model prediction for PHD2 knock-down, by measuring HIF-1α accumulation in 1% O_2_ in HeLa cells lacking PHD2 expression (stable shPHD2). The PHD2 knock-down was validated by Western blot (Fig. 6*D*) and no compensation by PHD3 was observed (Fig. 6*D*). Moreover, no strong overstabilization of HIF-1α was observed in normoxia or hypoxia compared with WT cells (Fig. 6*E*). This disagreed with the model prediction, which showed higher HIF levels in normoxia and hypoxia in the absence of PHD2. However, this could be explained by the differences observed between short-term and long-term knock-down (siPHD2 and shPHD2) previously discussed by Berra *et al.* (23). In hypoxia, HIF-1α dynamics were clearly different from those observed in wild type (WT) HeLa (Western blot Fig. 6*F* and imaging in Fig. 6*G*). We observed in most of the cases an accumulation of HIF-1α, which had either a long duration or did not show any noticeable decrease during the experiment. This was specific to PHD2 knock-down and was not observed in the case of PHD3 knock-down (Fig. 6, *H* and *I*).

##### Role of HIF Dynamics Controlled by PHD2 on Cell Survival

**FIGURE 7.**
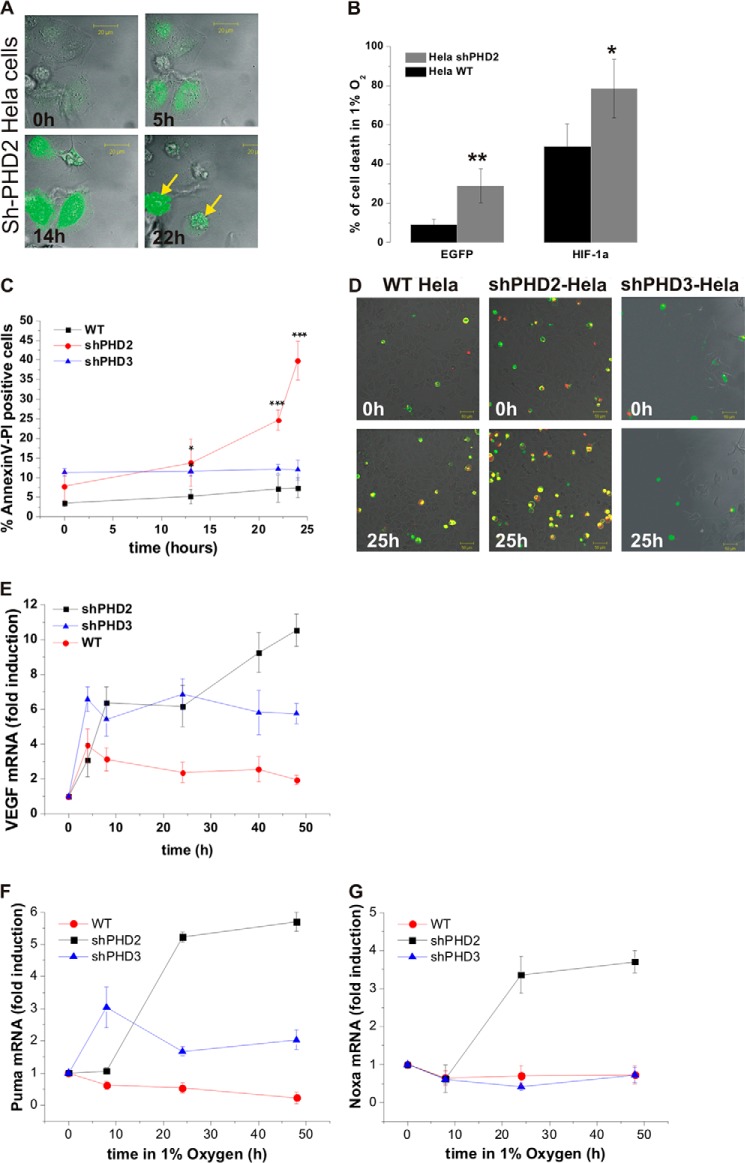
*A*, pictures of a representative field of sh-PHD2 cells expressing HIF-1α-EGFP at the indicated times in hypoxia show cell death associated with high and long lasting levels of HIF-1α. *B,* the percentage of transfected sh-PHD2 or WT HeLa cells dying within the 20 h of an imaging experiment was calculated for control cells transfected with either an empty EGFP plasmid or with HIF-1α-EGFP in normoxia and hypoxia (*n* = 40 cells/conditions). *C* and *D*, HeLa cells (WT, shPHD2 or shPHD3 lines) were imaged simultaneously using a 4-compartment glass bottom dish (Greiner). They were labeled with Annexin V-FITC (*green*) and PI (*red*) 10 min before imaging. Cells were imaged for 2 h in normoxia prior to the switch to 1% O_2_. Images were recorded every 15 min for 24 h. The number of apoptotic cells (Annexin labeling preceding the PI labeling) was counted out of the total number of cells and plotted (*C*). A typical field of cells at several time points is shown (*D*). *E–G*, WT, HeLa cells, or HeLa cells expressing shPHD2 or shPHD3, were cultured in 1% O_2_ for the indicated time points. *E,* VEGF mRNA levels were assessed by qPCR. *F*, PUMA mRNA levels were assessed by qPCR. *G,* Noxa mRNA were assessed by qPCR. *E–G*, the plots represent the average ± S.D. of triplicate samples from a representative experiment. The experiments were performed 4 times. Results are the mean of three independent experiments ± S.E. ***, **, and * indicate statistical difference with *p* < 0,001, *p* < 0.01, and *p* < 0.05, respectively.

We observed a very high level of cell death in hypoxic cells up-regulating HIF-1α-EGFP in the shPHD2 cell line (70%) compared with WT HeLa (40%) (Fig. 7, *A* and *B*). Apoptotic cell death was further quantified using Annexin V-PI labeling over a time course of hypoxia in WT and shPHD2/shPHD3 cells. Double labeling of Annexin V and PI was observed in 38% of shPHD2 cells after 24 h exposure to 1% O_2_ *versus* 7 and 12% in WT and shPHD3 cells, respectively (Fig. 7, *C* and *D*). Consequently, shPHD2 cells could not be tracked for a very long hypoxic period and the stability of the HIF up-regulation could not be studied. The consequences of the observed altered HIF-1α dynamics were investigated at the transcriptional level on a well defined HIF target gene. VEGF, showed a more sustained expression in shPHD2 cells compared with WT or shPHD3 cells (Fig. 7*E*), confirming a direct functional effect on HIF target genes of the PHD2 silencing. Because HIF has previously been demonstrated to interact with the mdm2 protein and affects p53 activity (11), we further investigated if the changes in HIF dynamics could affect p53 activity and hence explain the observed cell death in hypoxia when PHD2 is silenced. The transcription of two classical p53 target genes involved in pro-apoptotic signaling were measured by qPCR over a time course of hypoxia in WT HeLa cells as wells as in shPHD2 and shPHD3 cells. Interestingly, Noxa and Puma mRNA were both significantly up-regulated in hypoxia in the shPHD2 cells and were only marginally transcribed or even down-regulated in the shPHD3 or WT cells (Fig. 7, *F* and *G*).

**FIGURE 8.**
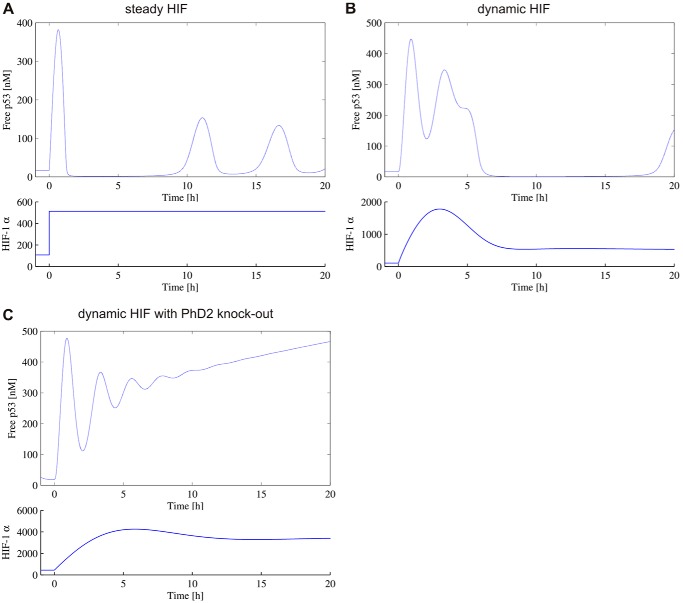
**Prediction of p53 dynamics following hypoxic switch.** *A*, the hypoxic switch at *t* = 0 is represented by an instantaneous switch in HIF from a low normoxic equilibrium level to a high hypoxic equilibrium level. The p53 levels are obtained by solving the HIF-dependent p53-MdM2 feedback model. *B* and *C,* the hypoxic switch drives transient HIF dynamics determined by the HIF-PHD model, which is coupled to the p53-MdM2 feedback model. Cells are WT (*B*) or sh-PHD2 (*C*).

To further explain the observed difference in p53 target gene transcription in the context of varying HIF dynamics, a previously described a p53 mathematical model coupled to HIF was used (see “Experimental Procedures”). When a hypoxic switch is represented by an instantaneous switch in HIF levels (Fig. 8*A*), in WT cells, p53 first displays a transitory peak and then establishes oscillatory dynamics as previously observed (12). However, when the dynamic nature of the HIF dynamics is explicitly included, the transitory behavior of p53 is markedly altered; displaying a double peak and delayed onset of oscillatory dynamics (Fig. 8*B*). Furthermore, when PHD2 is silenced, p53 displays sustained high levels (Fig. 8*C*). This is an example of how taking into account real protein dynamics instead of steady states might affect model coupling and could be applied to other systems than the p53 coupling used here. For example, the model coupling HIF with NO homeostasis also used steady state levels (24).

## DISCUSSION

Depending on the physiopathological context, cells experiencing hypoxia will be exposed to oxygen levels that vary in amplitude, duration (acute or chronic hypoxia), and possible preconditioning (25). In a situation such as ischemia (acute and strong hypoxia), HIF-1α activity has been associated with cell death (26), whereas in solid tumors it is associated with cell survival and proliferation (27). These diverse contexts and cell fate might be due to specific HIF accumulation profiles and subsequent differential binding to other partners and it is, therefore, important to understand the consequences of the variation of HIF timing to inform future therapeutic strategies aimed at controlling HIF activity. We have shown that in conditions where transient HIF accumulation was lost, there was a high level of cell death, pointing to the importance of elucidating which component of the HIF signaling is the guardian of its timing. Using a mathematical model built on single cell imaging data, we predicted that HIF-1α dynamics will display a range of kinetics depending on the hypoxic situation and that it is PHD2 that is specifically involved in the negative feedback responsible for pulsatile HIF levels.

### 

#### 

##### Single Cell Imaging of HIF: Transiency and Heterogeneity

Using live cell imaging, we observed a range of dynamics of HIF nuclear accumulation, including fast and slow kinetics, single and multiple peaks. Although heterogeneity is a common feature of cellular processes (22, 28), this could not be detected using bulk cell analysis approaches and has never been described previously in this system. In the case of the oxygen dependent signaling, inter-cellular heterogeneity may be generated by extrinsic noise such as NO or iron levels, as well as intrinsic noise from transcription (29). We also observed transient HIF accumulation in normoxia (Fig. 1*D*) and spontaneous peaks of transcriptional activity (not shown), which agrees with the previous suggestion of oxygen-independent mechanisms of HIF stabilization (18, 30).

##### HIF Dynamics: Role of Negative Feedback Loops and Their Mathematical Modeling

Existing HIF models (31–33) have focused on how equilibrium levels of HIF are a function of oxygen levels, in particular probing the possibility of a switch-like behavior in HIF levels in response to hypoxia. Despite being formulated as dynamic models, these previous models have typically focused on the static states of the system. However, the single-cell dynamic data presented here clearly demonstrate that in response to hypoxia, many cells undergo rapid and large amplitude transient dynamics in nuclear HIF, before returning to equilibrium levels, which are comparable with the levels found in normoxic conditions. The model proposed here is highly idealized in that they only consider the dynamics of HIF and the PHD proteins. This simple model might be integrated, in the future, in a recently published model based on HIF transcriptional activity (34). However, even our 2-component model, with the generic PHD, was able to fit a range of the single cell dynamic data and provided a tool to assess the sensitivity of the different model parameters via consideration of a median solution of the system. Furthermore, by expanding the model to consider separately PHD1, -2 and -3, we were able to test computationally potential specific roles of the PHDs in the control of HIF timing, and demonstrate non-redundancy between PHD1, -2, and -3. We were also able to examine downstream effects by coupling the simple HIF-PHD model to existing biochemical models. Specifically here we coupled HIF to a model of the p53-Mdm2 feedback loop. We were able to demonstrate the importance of capturing not only equilibrium levels of HIF in normoxia and hypoxia, but also the transient dynamics. Specifically we demonstrated that the overshoot HIF levels observed in transient dynamics lead to higher transitory levels of p53, and a delay in the onset of p53 oscillatory behavior. Furthermore, we determined *in silico* the HIF-mediated role that PHD2 has in regulating p53 dynamics, explicitly demonstrating that in shPHD2 cells, p53 displays sustained high levels suggesting apoptotic activity.

##### Role of the PHD2 Negative Feedback Loop on HIF Timing and Cell Fate

Berra *et al.* (23) addressed the question of the multiplicity of the PHDs relative to HIF-1α and showed, by silencing each PHD isoform individually, that only PHD2 controlled the steady state levels of HIF-1α in HeLa cells and other human cells. By extending a simple mathematical model of the HIF-PHD negative feedback loop it was possible to separate the 3 different PHDs on the basis of their levels, stability, and induction. This pointed to a strong role of PHD2 in the control of HIF nuclear accumulation in hypoxia. It is, however, possible that at other O_2_ levels, *e.g.* mild hypoxia or anoxia, the role of the other PHDs will also play an important role and hence contribute to HIF dynamics. The consequences of long lasting HIF-1α levels were observed on VEGF transcription dynamics (Fig. 7*E*), and they are likely to also directly affect HIF-binding proteins such as mdm2 (11), hence altering cell survival in hypoxia. The cell death induced by hypoxia in cells in which PHD2 was silenced (Fig. 7, *A–D*) was in line with the protective role of PHD2 in gliomas against hypoxia-induced tumor cell death (35) and could well represent a new molecular target for cancer drugs. Taken together, our data point to a major role for PHD2 compared with PHD3 in the negative feedback regulation of HIF-1α dynamics, although we cannot rule out the presence of other, non PHD-dependent, mechanisms, as previously suggested (36–39). The potential feedbacks, both oxygen-dependent and -independent, and how they affect HIF-α levels and activity will have to be further investigated.

Cells can experience hypoxia in a wide range of physiological and pathological contexts, where HIF activity is up-regulated and associated with different cell fates. The decision between survival/death, proliferation/cell cycle arrest in hypoxia is likely due to differential gene expression, as well as HIF binding to key proteins involved in these mechanisms (*e.g.* mdm2/p53). In conclusion, we have shown here that one way to trigger hypoxic death is to have uncontrolled high and long lasting HIF levels, and that an important role of PHD2 is to keep HIF on time. Our results show that HIF dynamics have an impact on cell fate through p53 transcriptional activity regulation, and mathematical modeling predictions points to differential p53 dynamics and levels depending on the HIF temporal profile, which will need to be fully investigated in the future.

## Supplementary Material

Supplemental Data

## Footnotes

8 The abbreviations used are:

HIF: hypoxia inducible factor
PHD: prolyl hydroxylase
PI: propidium iodide
EGFP: enhanced green fluorescent protein
HRE: hypoxia response element
ODD: oxygen-dependent domain
qPCR: quantitative PCR.
