# Supplementary material for: Tight Control of Hypoxia-inducible Factor-α Transient Dynamics Is Essential for Cell Survival in Hypoxia
Source: J Biol Chem. 2014 Jan 6;289(9):5549–64. doi: 10.1074/jbc.M113.500405 (PMC3937633; doi:10.1074/jbc.M113.500405)
Supplement: Supplemental Data [file supp_289_9_5549__index.html]

Tight control of Hypoxia Inducible Factor (HIF)-alpha transient dynamics is essential for cell survival in hypoxia — Tight Control of Hypoxia-inducible Factor-α Transient Dynamics Is Essential for Cell Survival in Hypoxia — HIF-α Dynamics and Mathematical Modeling — Supplemental Data 

# Tight Control of Hypoxia-inducible Factor-α Transient Dynamics Is Essential for Cell Survival in Hypoxia

## Supplemental Data

**Files in this Data Supplement:**

- supplemental figure S1 (.pdf, 1.7 MB) - Computational fit of single cell imaging traces.
- movie 1 (.avi, 7.7 MB) - HeLa cells were plated on a glass bottom dish and transfected with HIF-1alpha-EGFP 24 hours before imaging. Cells were then placed onto the microscope stage and imaged every 5 min as indicated in the main manuscript. Cells were initially imaged at 20.8%O2 for several frames before switching to 1%O2 for 20h. The movie is accelerated to 30 frames/sec.
- movie 2 (.avi, 2.8 MB) - HeLa cells were plated on a glass bottom dish and transfected with EGFP-HIF-2alpha 24 hours before imaging. Cells were then placed onto the microscope stage and imaged every 5 min as indicated in the main manuscript. Cells were initially imaged at 20.8%O2 for several frames before switching to 1%O2 for 15h. The movie is accelerated to 30 frames/sec.
