# Supplementary material for: Tight Control of Hypoxia-inducible Factor-α Transient Dynamics Is Essential for Cell Survival in Hypoxia
Source: J Biol Chem. 2014 Jan 6;289(9):5549–64. doi: 10.1074/jbc.M113.500405 (PMC3937633; doi:10.1074/jbc.M113.500405)
Supplement: Supplemental Data [file supp_M113.500405_jbc.M113.500405-1.pdf]

Supplemental material S1

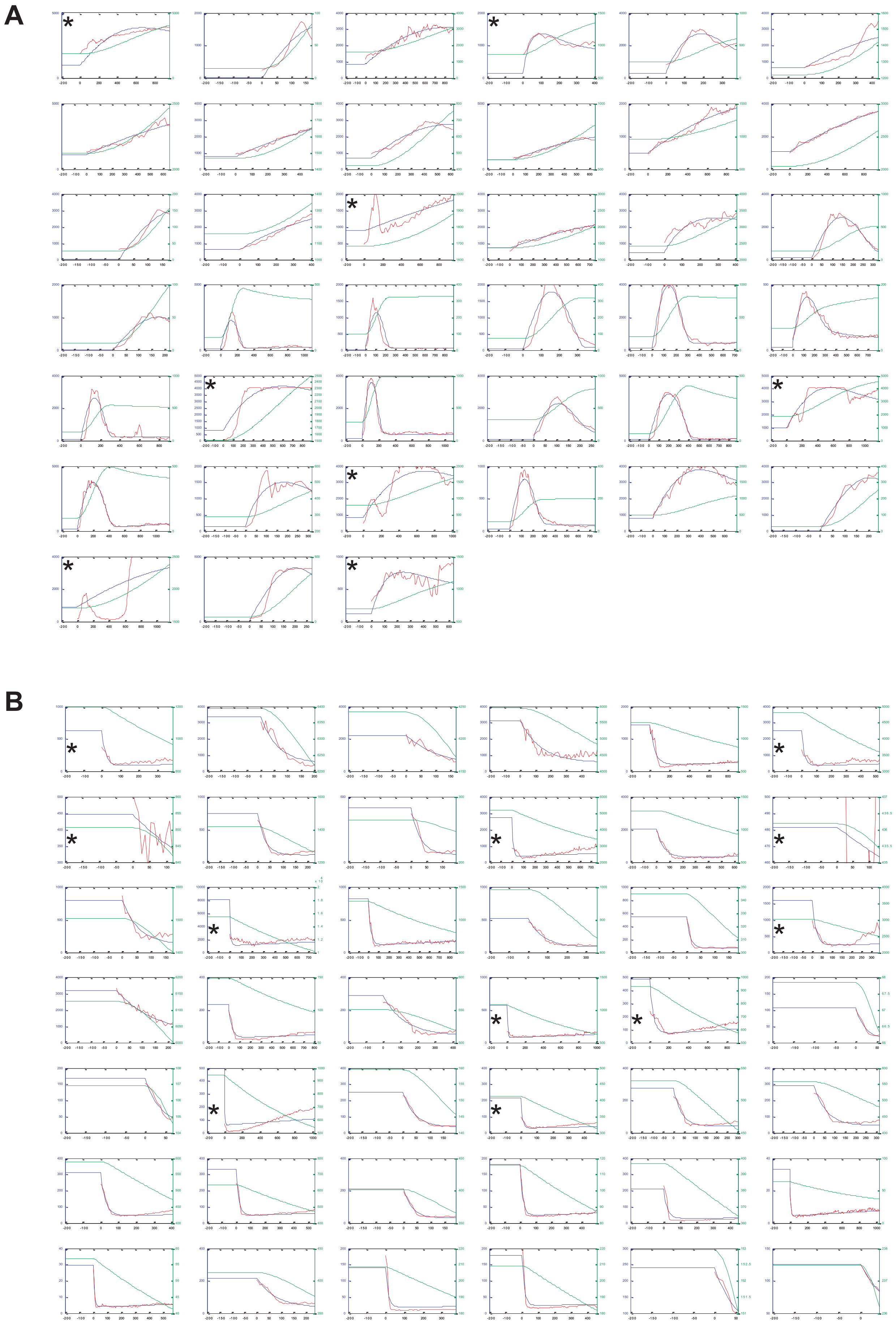

Fig S1: Computational fit of single cell imaging traces.
Single cells traces of HIF dynamics (blue line) that were fitted computationally using the model described in Fig 2 of the main manuscript (HIF, red line; PHD, green line). The * highlight the cells that could not be fitted appropriately with the 2 component model (see materiel and methods section) **A.** Cells are initially at equilibrium in normoxia (h=1) and are de-oxygenated into hypoxia (h=0.14) at t=0. **B.** Cells are initially at equilibrium in hypoxia (h=0.14) and are re-oxygenated back into normoxia (h=1) at t=0.

Supplemental movies

movie 1: HeLa cells were plated on a glass bottom dish and transfected with HIF-1α-EGFP 24h hours before imaging . Cells were then placed onto the microscope stage and imaged every 5 min as indicated in the main manuscript. Cells were initially imaged at 20.8%O_2 for several frames before switching to 1%O_2 for 20h. The movie is accelerated to 30 frames/sec.

movie 2: HeLa cells were plated on a glass bottom dish and transfected with EGFP-HIF-2α 24h hours before imaging . Cells were then placed onto the microscope stage and imaged every 5 min as indicated in the main manuscript. Cells were initially imaged at 20.8%O_2 for several frames before switching to 1%O_2 for 15h. The movie is accelerated to 30 frames/sec.
